# The Role of Immunohistochemistry in the Differential Diagnosis between Intrahepatic Cholangiocarcinoma, Hepatocellular Carcinoma and Liver Metastasis, as Well as Its Prognostic Value

**DOI:** 10.3390/diagnostics13091542

**Published:** 2023-04-25

**Authors:** Lavinia Patricia Mocan, Ioana Rusu, Carmen Stanca Melincovici, Bianca Adina Boșca, Tudor Mocan, Rareș Crăciun, Zeno Spârchez, Maria Iacobescu, Carmen Mihaela Mihu

**Affiliations:** 1 Department of Histology, “Iuliu Hațieganu” University of Medicine and Pharmacy, 400349 Cluj-Napoca, Romania; 2 Department of Pathology, “Prof. Dr. Octavian Fodor” Regional Institute of Gastroenterology and Hepatology, 400162 Cluj-Napoca, Romania; 3 UBBMed Department, Babeș-Balyai University, 400347 Cluj-Napoca, Romania; 4 Department of Gastroenterology, “Prof. Dr. Octavian Fodor” Regional Institute of Gastroenterology and Hepatology, 400162 Cluj-Napoca, Romania; 5 3rd Medical Department, “Iuliu Hațieganu” University of Medicine and Pharmacy, 400162 Cluj-Napoca, Romania; 6 Department of Proteomics and Metabolomics, MedFUTURE Research Center for Advanced Medicine, “Iuliu Hațieganu” University of Medicine and Pharmacy, 400349 Cluj-Napoca, Romania

**Keywords:** intrahepatic cholangiocarcinoma, hepatocellular carcinoma, secondary tumor, needle biopsy, immunohistochemistry, CK7, intratumoral immune cells

## Abstract

Intrahepatic cholangiocarcinoma (iCCA) is the second most frequent primary hepatic malignant tumor, after hepatocellular carcinoma (HCC). Its incidence has risen worldwide, yet the only potentially curative treatment, surgical resection, is seldom applicable, and the median overall survival remains extremely low. So far, there are no personalized therapy regimens. This study investigated whether routine immunohistochemical stains have diagnostic and/or prognostic value in iCCA. Clinical, imaging, and pathology data were retrospectively gathered for patients diagnosed with iCCA, HCC, or liver metastases assessed using liver needle biopsies. Three study groups with an equal number of cases (*n* = 65) were formed. In the iCCA group, CK19, CA19-9, CK7, and CEA demonstrated the highest sensitivities (100%, 100%, 93.7%, and 82.6%, respectively). The most relevant stains used for diagnosing HCCs were Glypican 3, CD34 (sinusoidal pattern), and Hep Par 1, with corresponding sensitivities of 100%, 100%, and 98.2%. The immunohistochemical panels for diagnosing metastatic tumors were chosen after correlating the clinical data and morphologic H&E aspects. Moderate/intensely positive CK7 expression and absent/low amount of intratumoral immune cells were favorable prognostic factors and correlated with increased overall survival in both the univariate analysis and the multivariate regression adjusted for age, existence of cirrhosis, number of tumors, and tumor differentiation.

## 1. Introduction

Intrahepatic cholangiocarcinoma (iCCA), a tumor derived from the biliary epithelium, is the second most frequent primary liver malignancy after hepatocellular carcinoma (HCC) and accounts for 10–20% of primary hepatic malignancies [1]. Conventionally, iCCA is located in the hepatic parenchyma, proximal to the left and right hepatic ducts [2]. Although less frequent than perihilar and distal cholangiocarcinoma, both classified as extrahepatic cholangiocarcinoma (eCCA), the incidence of iCCA is rising worldwide at a much greater rate compared to the incidence of eCCA, with a striking difference of a 350% vs. 20% increase [3,4].

Most risk factors for iCCA are associated with chronic liver inflammation: primary sclerosing cholangitis, hepatolithiasis, bile duct cysts and malformations, and liver flukes. The latter account for the development of most cholangiocarcinoma cases in endemic areas [5] but can also sporadically occur in Caucasian patients. Some authors include hepatitis B virus (HBV) and hepatitis C virus (HCV), chronic viral hepatitis, cirrhosis, non-alcoholic fatty liver disease, obesity, and diabetes among the risk factors [6,7,8]. Typically, both iCCA and HCC occur in the setting of chronic liver disease. In such cases, serum liver function tests, serology for viral hepatitis, alpha-fetoprotein (AFP), carcinoembryonic antigen (CEA), carbohydrate antigen 19-9 (CA19-9), and imaging studies with tumor characterization are part of the initial diagnostic workup.

In particular, imaging tests have a pivotal role in the diagnostic process. Differently from any other cancer entity, the diagnosis of HCC can be made based only on imaging if the hallmarks of HCC are present: arterial phase hyperenhancement (APHE), with washout in the portal venous or delayed phases on CT and MRI, using extracellular contrast agents or gadobenate dimeglumine; APHE with washout in the portal venous phase on MRI using gadoxetic acid; and APHE with late-onset (>60 s) washout of mild intensity on CEUS [9]. In the context of compensated advanced chronic liver disease (cACLD) and in the absence of non-invasive criteria, a liver tumor has the same probability of being either HCC or iCCA, and a liver biopsy (LB) is mandatory for a definite diagnosis [10]. Among the different HCC histological subtypes, steatohepatitic HCC, scirrhous HCC, and the macrotrabecular massive HCC do not display typical HCC features on imaging [11]. In the clinical context of a patient with cACLD, one should rarely consider a secondary liver tumor, since this situation is infrequent in clinical practice. According to one metanalysis, only 1.7% of liver masses from 1453 cirrhotic livers were metastases [12]. One should, however, bear in mind the possibility of the association between non-Hodgkin B-cell lymphoma and hepatitis C virus infection [13]. On the other hand, when cACLD is not present, a liver nodule has the same chance of being an HCC, an iCCA, or a secondary tumor. Clinical data and imaging tools can be helpful in this setting, but the final diagnosis relies on LB. For example, a prior history of malignant disease in a patient with liver nodules might hint at secondary tumors, or sectional imaging might incidentally reveal the presence of the primary tumor, and depending on the location, endoscopy might confirm the final diagnosis.

As seen above, LB is necessary in some clinical scenarios. However, assessing whether a LB is necessary in a case-by-case manner is essential, given that it is an invasive procedure that exposes the patient to risks such as bleeding and seeding [14]. LB only offers a small tumor fragment, while pressuring the pathologist to extract maximal information. Differential diagnosis between iCCA, HCC, and liver metastases is sometimes not straightforward. Moreover, discriminating between the three types of tumor using only the basic hematoxylin and eosin (H&E) stain can be difficult. One can perform a limited number of immunohistochemical stains on such a small sample. Therefore, it is vital to know the complete clinical history and only afterward choose the correct immunohistochemical markers. Moreover, the immunohistochemical spectrum has tremendous potential for clinical practice, since multiple markers can have diagnostic, theranostic, or prognostic power. Nevertheless, prognostic biomarkers in liver cancer are a necessity. Current iCCA prognostic predictors include large tumor size (tumor > 5 cm, as stated in the current 8th edition of the AJCC staging system), multiple tumors, vascular invasion, perineural infiltration, and positive regional lymph nodes (N1) [15,16]. However, the evidence supporting these predictors is not unanimous, as not all authors reached a consensus in extensive multicentric studies. One striking example is related to tumor size, which was associated with survival only in univariate analysis in a large multi-institutional study that included 449 iCCA resection specimens. This correlation was not maintained in the multivariate regression model [17]. Some immunohistochemical markers already used in daily practice to diagnose HCC or iCCA might also have prognostic potential.

Therefore, our primary aim was to investigate which markers can aid the discrimination between the three entities, based on the experience of a tertiary hepatobiliary healthcare facility. Our second aim was to investigate whether certain immunohistochemical stains have a prognostic role correlating with patient survival and whether other readily-available pathological parameters could represent prognostic markers for iCCA or HCC.

## 2. Materials and Methods

### 2.1. Case Selection

Three Caucasian cohorts, including a matching number of cases (*n* = 65) with pathologic diagnosis of iCCA (group 1), HCC (group 2), and metastatic hepatic tumors (group 3), established with a needle biopsy performed during 2014–2021 were retrospectively selected from the hospital’s database. Groups 1 and 2 only included patients with advanced, unresectable tumors. We decided to only include patients with advanced HCC or iCCA because (a) we rarely perform LB in patients with resectable HCC or iCCA at our center; (b) the majority of iCCA are diagnosed at an advanced stage, and we decided to compare them with advanced HCC (and not with early HCC) and, therefore, to avoid potential bias; and (c) the patients with liver metastases were already at an advanced stage, and therefore we wanted to avoid further bias. Clinical data and imaging studies were further analyzed for each patient, to ensure correct assignment to the study groups. Alive/dead status and the date of death were obtained in December 2022, and overall survival (OS) (from initial diagnosis until death) was determined. For the patients in group 3, an additional survival period (from the secondary hepatic tumor diagnosis to the time of death) was calculated. Data from patients alive at the end of follow-up were censored in the statistical analysis. In total, 15 patients were excluded: six patients with metachronous iCCA and HCC, two patients with combined hepatocellular–cholangiocarcinoma, and seven cases where the diagnosis was established without the use of immunohistochemical stains.

### 2.2. Data Gathering and Interpretation of Pathology Slides

Clinical, laboratory, and imaging characteristics were recorded for each case. They included general demographic parameters, relevant clinical characteristics, associated diseases, nonspecific serum tumoral markers, number of tumors (solitary or multifocal), and tumor size. The pathological parameters available in small biopsies were as follows: final diagnosis (iCCA, HCC, or histologic type of metastasis along with primary site), tumor differentiation (well, moderate, or weak), and intratumoral immune infiltrate (absent, weak, moderate, or abundant). The immunohistochemical stains used for diagnostic purposes were performed on 3 μm tissue sections, using completely automated systems (Leica Bond-Max Immunostainer; Leica Biosystems, Nussloch, Germany), according to the manufacturer’s instructions. Two pathologists reevaluated all slides blind to the clinical data, to ensure uniformity of stain intensity interpretation. Stains were scored using a four-tier system: negative, weakly positive, medium positive, and intensely positive. Pathologists requested all immunohistochemical stains made during the initial case evaluation, for diagnostic purposes.

### 2.3. Statistical Analysis

Categorical data were presented as counts and percentages. Comparisons of categorical data were performed using a Chi-square or Fisher’s exact test in case of low expected frequencies. Continuous normally distributed data were reported as means and standard deviations, and skewed data as medians and quartiles. Comparisons of continuous skewed data were performed with a Wilcoxon rank sum test. Spearman’s correlation coefficient and its associated statistical test assessed the correlation between continuous skewed data. The OS was defined as the time from diagnosis until death or the study end date (December 2022). Survival data were graphically presented using the Kaplan–Meier method. Univariate proportional Cox regression verified the relationship between various immunohistochemical markers and survival. To confirm that these relations were not spurious, we further added known predictors of survival as adjustment variables in the multivariate Cox regression models. The proportional hazard assumption was checked with a formal statistical test for all these models, while the linear functional form for continuous variables was checked using model residual plot inspection. For multivariate models, multicollinearity was assessed with variance inflation factors. The two-tailed *p*-value was computed for all statistical tests, and the results were considered statistically significant for values below 0.05. All analyses were computed using the R environment for statistical computing and graphics (R Foundation for Statistical Computing, Vienna, Austria), version 3.6.3, R Core Team. R: A Language and Environment for Statistical Computing (Internet), Vienna, Austria; 2019.

### 2.4. Ethics Committee

Approvals from the Ethics Committees of both “Iuliu Hațieganu” University of Medicine and Pharmacy (34/13 December 2021) and “Octavian Fodor” Regional Institute of Gastroenterology and Hepatology (165/9 December 2021) were obtained. All biopsies analyzed in this study were performed for diagnostic purposes; consequently, patient consent was waived.

## 3. Results

### 3.1. General Findings

A total of 195 patients were included in the study. The baseline patient characteristics are depicted in Table 1.

Most patients with metastatic liver disease (group 3) had multifocal lesions (92.3%). This was also the case in primary tumors, since more than half (60% iCCAs and 56.92% HCCs) had multiple tumors. Liver metastases originated from the following primary tumors (in descending order): colorectal carcinomas (25 cases, 38.45%), neuroendocrine carcinomas (10 cases, 15.38% with pancreatic, pulmonary or unassigned primary location), pancreatic ductal adenocarcinomas (9 cases, 13.85%), invasive breast carcinomas (7 cases, 10.78%), and gastric adenocarcinomas (3 cases, 4.61%).

### 3.2. Immunohistochemical Markers Expressed in HCC, iCCA, and Liver Metastases

The most relevant antibodies for iCCA were CK19, CA19-9, CK7, and CEA; the corresponding sensitivity of each marker was 100%, 100%, 93.7%, and 82.6%, respectively. The most relevant antibodies for HCC were Glypican 3, CD34 (with sinusoidal pattern), and Hep Par 1; the corresponding sensitivity of each marker was 100%, 100%, and 98.2%, respectively. One case with iCCA tested positive for Hep Par 1 (low intensity), and one iCCA tested positive for Gypican 3 (low intensity). Three cases with iCCA expressed CD34 but none showed a sinusoidal pattern. Four cases from the iCCA group were CK20-positive, but three of the four only expressed a weak intensity, while the other expressed a moderate intensity. From the HCC cohort, only one case was positive for CK7 (weak intensity), and three cases were CK19-positive, all with weak intensity.

The most used markers in liver metastases were CDX2, CK7, CK20, and CK AE1/AE3. No case from the liver metastases group tested positive for Hep Par 1 or Glypican 3. Four cases from iCCA were CK20-positive (three of four cases showed weak intensity and one moderate intensity). In the iCCA group, 11 cases tested positive for CDX2, while ten had weak intensity and only one had moderate intensity. The most important and highly expressed immunohistochemical markers in each cancer entity (HCC, iCCA, and liver metastases) are depicted in Table 2.

### 3.3. Prognotic Markers of iCCA

**Figure 1:**
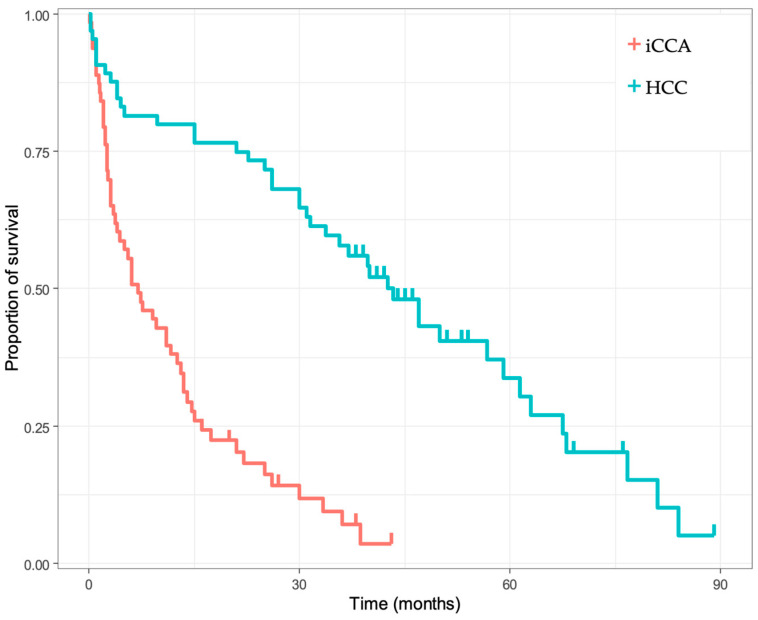
Kaplan–Meier survival analysis regarding the pathology-confirmed diagnosis of intrahepatic cholangiocarcinoma (iCCA) versus hepatocellular carcinoma (HCC); *p* < 0.001 (log-rank test).

The subsequent focus was to identify histological or immunohistochemical-based prognostic biomarkers. We first compared the OS between the two most frequent primary liver cancers. As shown in Figure 1, patients diagnosed with iCCA had a strikingly lower OS than HCC patients (months, interquartile range): 38.1 (27.81–52.19), 18.31 (10.58–31.69), 7.12 (2.54–19.97), and 3.56 (0.63–20.03) for the iCCA group; compared to 79.91 (70.72–90.3), 73.25 (63.11–85.03), 57.85 (46.67–71.72), and 43.2 (31.77–58.76) for the HCC group at 12, 24, 36, and 48 months, respectively, *p* < 0.001 (log-rank test).

**Figure 2:**
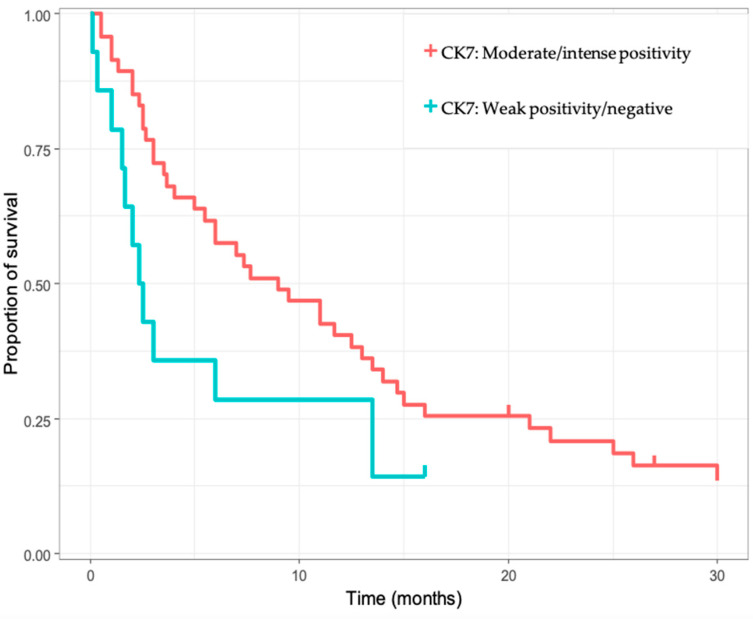
Kaplan–Meier survival analysis for intrahepatic cholangiocarcinoma (iCCA) cases, regarding CK7 immunoexpression.

**Figure 3:**
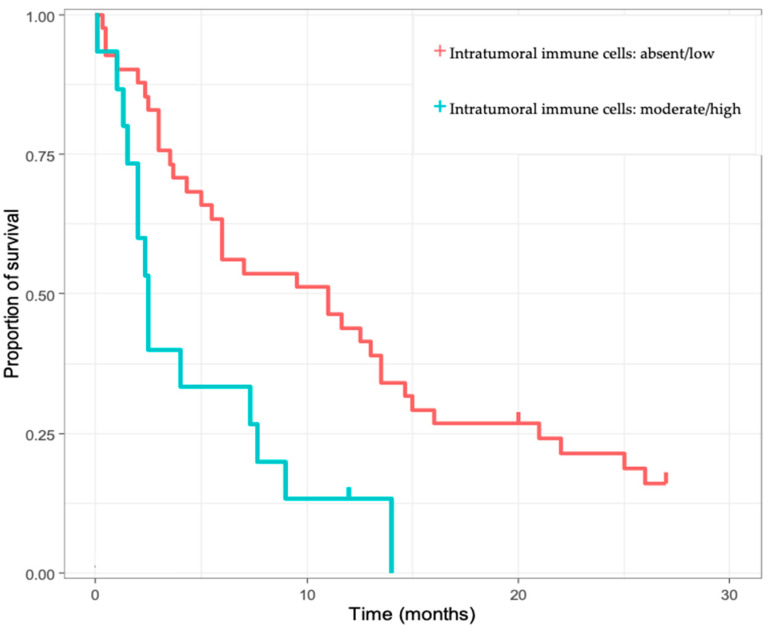
Kaplan–Meier survival analysis for intrahepatic cholangiocarcinoma (iCCA) cases based on the amount of intratumoral immune cells.

Next, we performed a univariate analysis, to search for prognostic biomarkers. Among the multiple biomarkers included in the analyses (tumor size, age, tumor number, tumor differentiation, tumor size, presence of cirrhosis, CDX2, CK19, CK7, pCEA, mCEA, CA19-9), only CK7 (Figure 2) and the presence of immune cell infiltrates (Figure 3) were correlated with OS (*p* = 0.016, *p* = 0.0028). Furthermore, both moderate/intense CK7 positivity and absence/low amount of immune cell infiltrate remained as positive prognostic biomarkers in the multivariate analysis (Table 3).

### 3.4. Prognostic Markers of HCC

The subsequent focus was to identify histological- or immunohistochemical-based prognostic markers. Therefore, we performed an univariate analysis. None of the multiple biomarkers included in the analyses (age, tumor number, intratumoral lymphocytes, liver cirrhosis, Hep Par 1, CD34, Glypican 3) reached statistical significance (*p* = 0.68, *p* = 0.22, *p* = 0.54, *p* = 0.60, *p* = 0.68, *p* = 0.79, and *p* = 0.53, respectively).

## 4. Discussion

### 4.1. Diagnostic Perspectives

#### 4.1.1. iCCA vs. HCC

Hepatocyte paraffin 1 (Hep Par 1) demonstrates the hepatocellular origin of tumor cells, dyes normal and neoplastic hepatocytes, and should be considered positive in cytoplasmic, diffuse, granular staining. Both the sensitivity and specificity of Hep Par 1 exceed 90% [18,19]. Similarly to in our study, where one iCCA case showed weak Hep Par 1 positivity, other authors reported Hep Par 1 positivity in small subsets of cholangiocarcinomas [18,19]. These data suggest that diagnosis of cholangiocarcinoma should not be ruled out solely based on Hep Par 1 positivity but it is highly unlikely in cases with high-intensity staining. Conversely, poorly differentiated HCCs can lose Hep Par 1 expression [20]. Moreover, small HCC needle biopsies can result in false-negative interpretations due to discontinuous staining. Hep Par 1 can show positivity in scarce hepatoid variants of gastrointestinal and pancreatic adenocarcinomas [21]. Our series had no Hep Par 1-positive cases among the metastatic tumors.

Glypican 3 is highly expressed in embryonal tissue and should be considered positive in cases with strong and diffuse cytoplasmic staining, with or without membranous staining. The sensitivity ranges between 53% and 100% in resection specimens with low values for well-differentiated HCCs, but percentages reach 100% in poorly differentiated tumors [22]. This particularity confers Glypican 3 a substantial discriminative value in poorly differentiated HCCs, since Hep Par 1 frequently loses expression in these scenarios. Sensitivity is lower in needle biopsies [23] and the specificity is also low, since Glypican 3 marks other hepatic or extrahepatic tumors, such as hepatoblastomas, ovarian clear cell carcinomas, testicular yolk sac tumors, choriocarcinomas, and specific subsets of melanomas and lung squamous cell carcinomas [24]. Glypican 3 discriminates well between HCC and cholangiocarcinoma (intrahepatic and extrahepatic), since its expression is downregulated in the latter [25]. In our study, all HCC cases (irrespective of their histologic differentiation) were Glypican 3-positive, while only one iCCA case showed weak Glypican 3 positivity.

CD34 marks sinusoidal capillarization in HCC, with uniform intensity and distribution, while normal sinusoidal endothelial cells are CD34-negative. In our study, all HCC cases demonstrated CD34 positivity with a sinusoidal pattern. Three iCCA cases were positive for CD34. Nevertheless, none showed a sinusoidal pattern.

Cytokeratin 7 (CK7) and Cytokeratin 20 (CK20) display various patterns in the epithelia throughout the human body. Hence pathologists frequently describe them in conjunction. CK7 is expressed in normal bile duct epithelia but not in hepatocytes. CK20 shows a variable expression, generally positive in extrahepatic bile duct tumors, including gall bladder carcinoma, but negative in both HCC and iCCA [26]. Our findings were in accordance with this. CK7 was utilized in 96.92% of iCCA cases, among which 59 cases (93.65%) were positive, while four cases (6.77%) were CK7-negative. A single HCC case was CK7-positive but showed weak intensity. CK20 was utilized in 41 iCCA cases (63.07%). Among these, only four cases (9.75%) were CK20-positive, showing a weak intensity in three cases and moderate intensity in one case.

Cytokeratin 19 (CK19) stains bile ducts in cirrhotic nodules and is generally CK19-negative in HCC. In a study by Durnez et al., 16% of HCC cases were CK19-positive [27]. In our study, three cases from the HCC group stained positive for CK19, all showing weak intensity.

Polyclonal carcinoembryonic antigen (pCEA) displays a canalicular staining pattern in HCC, with a sensitivity ranging between 50 and 96%, with higher percentages in well- and moderately differentiated tumors. However, it shows a diffuse cytoplasmic and luminal pattern in iCCA and part of metastatic tumors [26,28]. Monoclonal carcinoembryonic antigen (mCEA) is usually positive in iCCA and negative in HCC [29]. We analyzed pCEA in 23 iCCA cases, among which 19 (82.61%) were positive.

Although not an immunohistochemical stain, Alcian blue can aid in distinguishing poorly differentiated HCC from iCCA, by highlighting mucus secretion within the cytoplasm of tumoral cells and thus confirming a glandular phenotype in the latter, but not in HCC [30].

#### 4.1.2. iCCA vs. Metastatic Tumors

In the metastatic tumor group, immunoassays were requested in concordance with existing clinical data, pursuing tissue-specific markers.

CDX2 is a transcription factor expressed in the small intestine and colon. It stains normal intestinal epithelium, hyperplastic colonic polyps [31], and colorectal adenocarcinoma. Consequently, it is the first choice and sometimes the only immunostaining required to confirm the clinical diagnosis, but it is a highly non-specific marker for colorectal adenocarcinoma. CDX2 marks 86–100% [32] well- and moderately-differentiated colorectal adenocarcinomas but is also immune-positive in intestinal metaplasia, wherever it occurs. Therefore, it can serve as a marker for intestinal differentiation. However, there is evidence that CDX2 is positive in subgroups of ovarian mucinous adenocarcinomas; 30% of cervical mucinous adenocarcinomas [33]; small intestine carcinoma; 36–70% of gastric adenocarcinomas, including signet ring cell adenocarcinomas, urothelial carcinoma, and pancreatic [34], ileal, and appendicular neuroendocrine tumors. CDX2 also stains over one-third (37.3%) of eCCAs and gall bladder carcinomas [35]. Thus, CDX2 is considered less specific than the CK7-negative/CK20-positive panel for colorectal carcinoma [36]. In our study, CDX2 was performed in 42 iCCA cases (64.61%), among which 11 cases (26.19%) were CDX2-positive. However, 10 out of 11 cases showed weak positivity, and one showed moderate positivity. In group 3, CDX2 staining was performed in all metastases with colorectal primaries. All cases (*n* = 25) showed CDX2 positivity. Among these, 24 cases demonstrated moderate- and high-intensity staining, while only one showed weak CDX2 staining. Among the pancreatic ductal adenocarcinoma cases, 71.42% demonstrated weak CDX2 positivity (80%) or moderate CDX2 positivity (20%).

A major shortcoming is the lack of reliable biomarkers for distinguishing iCCA from gastric and pancreatic adenocarcinomas and between iCCA, eCCA, and gall bladder carcinomas. Indeed some markers are undergoing evaluation [37,38] but have yet to reach clinical practice. Until then, the clinical context and the proper use of paraclinical tools are crucial. For instance, when discriminating between iCCA and gastric cancer, the epistemologically sound approach should always include an upper gastrointestinal endoscopy to settle diagnostic doubts. Following the same rationale, discriminating between iCCA and pancreatic adenocarcinoma should, at least in theory, be facilitated by imaging tools to pinpoint the primary tumor.

Finally, if the immune profile is extremely ambiguous or inconclusive, we recommend returning to the H&E morphology.

### 4.2. Prognostic Perspectives

Our results confirmed that advanced iCCA has a worse prognosis when compared to advanced HCC, which is concordant with existing literature data. This statement further reinforces the importance of accurate early diagnosis. Several studies have focused on identifying biomarkers for iCCA patients using various omics methods [39]. However, little attention, if any, has been given to developing immunohistochemical-based biomarkers for the prognoses that are already available in clinical practice and with which the pathologist has had time to familiarize. A meta-analysis that evaluated 77 different proteins within 73 research studies listed five immunohistochemical markers associated with patient outcome: EGFR, MUC1, MUC4, p27, and fascein [40]. Among these, only MUC1 (also known as EMA) has entered routine practice and promises to ensure reproducibility in large case series.

A recent study conducted by He et al. demonstrated a significant association between the post-surgery survival of iCCA patients and two immunohistochemical markers: while SATB1 indicated poor survival (median survival of 122 days vs. 347 days in SATB1-negative cases, *p* = 0.04), Villin-positive cases were associated with better OS, with direct correlation with Villin intensity (*p* = 0.002). This study recommended CK7 assessment in iCCA cases, since it was negatively correlated with lymphatic metastasis in their case series [41]. An interesting study conducted by Yeh et al. in Taipei validated C-reactive protein (CRP) as a highly performant diagnostic marker for iCCA, with a 93.3% sensitivity and an 88.2% specificity. CRP also correlated with better OS (*p* = 0.002) and longer postoperative recurrence-free time (*p* = 0.032) [42].

Our study showed that CK7-positive iCCA patients had a better OS. Until now, only one study has evaluated the prognostic potential of CK7 and CK19 in surgically resected iCCA patients. Based on the mARN levels of both CK7 and CK19, the authors showed that the CK7-positive/CK19-positive index was an independent adverse prognostic factor for survival in iCCA [43]. In addition, our study has shown that the presence of intratumoral immune cells bears a negative prognostic significance. While the results from this dataset appear to contrast with a previously published report from our study group, in which PD-L1-positive intratumoral cells had a positive predictive impact, the difference is more nuanced and resides in PD-L1 staining, the types of immune cell studied, and population selection (early vs. advanced disease) [44]. For a further expansion on this topic, one systematic review discussed the discrepancy between intratumoral immune cells and the prognosis of iCCA patients (some studies describe intratumoral immune cells as positive prognostic markers, while others as negative prognostic markers) [45]. The type of lymphocytes infiltrating iCCA is also important: Dong Liu et al. compared CD8-positive with Foxp3-positive lymphocytes, the latter having a positive prognostic value [45]. We did not evaluate the type of intratumoral lymphocytes in our study. However, our findings are significant, since tumor-infiltrating lymphocytes might correlate with the response to durvalumab, a checkpoint inhibitor recently approved for the systemic treatment of iCCA, based on a presumption extrapolated from HCC [46].

Our study has several limitations. First, it was a retrospective study, with all the limitations derived from this. The study only included advanced epithelial primary liver tumors and did not analyze other malignant liver tumors, such as hemangioendothelioma, lymphoma, or angiosarcoma. However, the three entities included represent the vast majority of those encountered in daily clinical practice. Second, we analyzed only the immunohistochemical markers routinely used for diagnostic purposes; therefore, other prognostic markers frequently analyzed in experimental settings could not be assessed. Third, a thorough analysis of the tumoral microenvironment was not an essential step in the study design. Hence, the reporting on tumor-infiltrating immune cells should be interpreted cautiously, since this represents only a quantitative estimate, with no in-depth reporting on the type of cells and expression.

Nonetheless, despite all the limitations, some important conclusions can be made. First, for a definite diagnosis, knowing the clinical context of each patient is mandatory. None of the immunohistochemical markers evaluated in our study showed a perfect delineation between the three cancer entities, and therefore one should only perform a LB when necessary. In some situations, LB is unnecessary (e.g., liver nodule with typical HCC features on imaging), while in others LB is not feasible (e.g., small tumors or tumors located in segment VI or VIII) [47]. Moreover, LB is an invasive procedure, which poses non-negligible risks, despite an overall safe profile [48]. Last, LB offers a limited tissue fragment, so one should maximize the amount of data extracted from it. Unfortunately, a biopsy cannot be repeated ad libitum, and the number of immunohistochemical stains per fragment is finite. Therefore, using a panel of carefully selected immunohistochemical markers can facilitate a less expensive and laborious final diagnosis.

## 5. Conclusions

Immunohistochemical stains should be assessed, first and foremost, in conjunction with morphology and clinical data. Nothing is black and white in microscopy, and immunohistochemistry is no exception. In liver tumors, as in other sites, immuno-histochemical panels remain superior to single colorations. Furthermore, apart from diagnosis, immunohistochemical studies can also provide prognostic information. Lastly, we strongly recommend mentioning both the presence and the amount of intratumoral immune infiltrate in routine pathologic reports.

## Figures and Tables

**Table 1:** Baseline characteristics of the study population.

Patient Characteristics	Intrahepatic Cholangiocarcinoma	Hepatocellular Carcinoma	Liver Secondary Tumors	*p* Value
Number of patients	65	65	65	ns
Clinical parameters				
Age				
Mean ± SD	64.42 ± 9.23	65.57 ± 6.49	63.06 ± 9.78	ns
Range	41–84	51–80	39–85	
Sex, *n* (%)				
Male	34 (52.3)	50 (76.92)	31 (47.69)	ns
Female	31 (47.69)	15 (23.08)	34 (52.3)	
Environment, *n* (%)				
Urban	35 (53.85)	45 (69.23)	50 (76.92)	0.0013
Rural	30 (46.15)	20 (30.77)	15 (23.08)	
Associated diseases, *n* (%)				
Obesity	6 (9.23)	8 (12.3)	9 (13.84)	ns
Diabetes mellitus	13 (20)	15 (23.07)	13 (20)	ns
Liver steatosis	16 (24.61)	15 (23.07)	13 (20)	ns
Chronic Hepatitis				
HBV	7 (10.77)	11 (16.92)	1 (1.53)	0.01
HCV	10 (15.38)	31 (47.69)	2 (3.07)	<0.001
Alcohol abuse	3 (4.61)	15 (23.07)	3 (4.61)	<0.001
Liver cirrhosis	12 (18.46)	53 (81.53)	4 (61.53)	<0.001
Ethanolic	3 (4.61)	14 (21.53)	1 (1.53)	<0.001
HBV	3 (4.61)	8 (12.3)	-	nc
HCV	5 (7.69)	29 (44.61)	1 (1.53)	<0.001
Autoimmune	1 (1.53)	-	-	ns
Metabolic	-	1 (1.53)	-	nc
Idiopathic	1 (1.53)	3 (4.61)	1 (1.53)	nc
Overall survival (months)				
Mean ± SD	9.25 ± 9.65	31.22 ± 24.9	31.85 ± 44.47	<0.001
Range	0.1–38.66	0.16–84	0.5–192	
Serum tumoral markers				
AFP	72.59 ± 139.51	95.74 ± 151.93	24.84 ± 90.93	nc
CEA	8.84 ± 13.32	3.65 ± 4.02	40.64 ± 58.23	nc
CA 19-9	202.14 ± 162.71	98.36 ± 95.12	146.84 ± 170.3	nc
Morphologic parameters				
Tumor size (cm)				
Mean ± SD	8.05 ± 3.58	5.45 ± 4.11	4.86 ± 3.81	<0.001
Range	0.6–16	1.3–19	0.5–18	
Number of tumors, *n* (%)				
Solitary	26 (40)	28 (43.07)	5 (7.7)	<0.001
Multiple	39 (60)	37 (56.92)	60 (92.3)	
Tumor differentiation, *n* (%)				
Good	12 (18.46)	6 (9.24)	16 (24.61)	<0.001
Moderate	23 (35.38)	45 (69.24)	17 (26.16)	
Poor	15 (23.07)	3 (4.62)	8 (12.31)	
N/A	14 (21.53)	11 (16.9)	24 (36.92)	
Tumor infiltrating lymphocytes count, *n* (%)				
Low	38 (58.46)	26 (40)	23 (35.38)	<0.001
Moderate	17 (26.15)	5 (7.69)	13 (20)	
Abundant	0	1 (1.54)	1 (1.54)	
Absent	3 (4.62)	0	0	
N/A	7 (10.77)	33 (50.77)	28 (43.08)	
Number of immunohistochemical stains used (Mean ± SD)	8 ± 3	4 ± 1.67	6 ± 3.62	nc

nc = not calculated due to low sample size, ns = not significant.

**Table 2:** The most common immunohistochemistry markers expressed in the different types of liver cancer.

Marker	iCCA	HCC	Liver Metastases	*p* Value
CK7, *n* (%) *	59 (93.7)	1 (14.3)	25 (80.6)	<0.001
CK19, *n* (%)	43 (100)	3 (37.5)	6 (54.5)	<0.001
CEA, *n* (%)	19 (82.6)	4 (44.4)	5 (50)	<0.001
CA19-9, *n* (%)	9 (100)	0	6 (100)	<0.001
Hep Par 1, *n* (%)	1 (3.3)	55 (98.2)	0	<0.001
Glypican 3, *n* (%)	1 (16.7)	44 (100)	0	<0.001
CD34, *n* (%)	3 (37.5)	54 (100)	0	<0.001
CDX2, *n* (%)	12 (28.6)	0	36 (94.7)	<0.001
CK20, *n* (%)	4 (9.8%)	0	18 (94.7)	<0.001

In blue—the most frequently expressed markers in intrahepatic cholangiocarcinoma; in red—the most frequently expressed markers in hepatocellular carcinoma; in green—the most frequently expressed markers in liver metastases from colorectal carcinoma; HCC = hepatocellular carcinoma, iCCA = intrahepatic cholangiocarcinoma; *n* = number, % = per cent; *p* = level of significance, CK = cytokeratin; CA = carcinogenic antigen; CEA = carcinoembryonic antigen; CD = cluster of differentiation; * a marker expressed in both iCCA and liver metastases from colorectal carcinoma.

**Table 3:** Univariate and multivariate analysis of overall survival in intrahepatic cholangiocarcinoma (iCCA) patients.

OS	Univariate Analysis			Multivariate Analysis		
	HR	95% CI	*p*	HR	95% CI	*p*
Age	0.97	0.94–1	0.076			
Tumor number (multiple vs. single)	1.42	0.82–2.46	0.208			
Liver cirrhosis (yes vs. no)	0.93	0.46–1.85	0.828			
Immune cell infiltrate (yes vs. no) *	2.68	1.38–5.2	0.004	3.64	1.67–7.9	0.001
Tumor differentiation **	1.11	0.56–2.18	0.771			
CDX2 (positive vs. negative)	1.84	0.88–3.85	0.108			
CK7 negative (yes vs. no) ***	1.82	0.92–3.6	0.087	2.42	1.1–5.33	0.028
CK19 negative (yes vs. no) ^#^	0.48	0.22–1.03	0.06			

* we compared intense and moderate with low or negative; ** we compared well-differentiated with moderate and poor differentiation; *** patients CK7-negative or with a weak staining were compared with moderate or intense staining; ^#^ patients CK19-negative or with a weak staining were compared with moderate or intense staining; *p* = level of significance; CI = confidence interval; HR = hazard ratio.

## Data Availability

The data presented in this study are available on request from the corresponding author.
